# Development, successes, and potential pitfalls of multidisciplinary chronic disease management clinics in a family health team: a qualitative study

**DOI:** 10.1186/s12875-023-02073-x

**Published:** 2023-06-20

**Authors:** Laura Brooks, Jacobi Elliott, Paul Stolee, Veronique Maria Boscart, Sarah Gimbel, Brittany Holisek, Jason Randle, George Albert Heckman

**Affiliations:** 1 University of Waterloo, Waterloo, ON Canada; 2 New Vision Family Health, Kitchener, Canada; 3 McMaster University, Hamilton, Canada; 4 Lakehead University, Thunder Bay, ON Canada; 5 Research Institute for Aging, 250 Laurelwood Drive, Waterloo, ON N2J 0E2 Canada

**Keywords:** Primary care, Chronic disease, Older adults, Frailty, Multidisciplinary teams

## Abstract

**Background:**

The creation of Family Health Teams in Ontario was intended to reconfigure primary care services to better meet the needs of an aging population, an increasing proportion of which is affected by frailty and multimorbidity. However, evaluations of family health teams have yielded mixed results.

**Methods:**

We conducted interviews with 22 health professionals affiliated or working with a well-established family health team in Southwest Ontario to understand how it approached the development of interprofessional chronic disease management programs, including successes and areas for improvement.

**Results:**

Qualitative analysis of the transcripts identified two primary themes: [1] Interprofessional team building and [2] Inadvertent creation of silos. Within the first theme, two subthemes were identified: (a) collegial learning and (b) informal and electronic communication.

**Conclusion:**

Emphasis on collegiality among professionals, rather than on more traditional hierarchical relationships and common workspaces, created opportunities for better informal communication and shared learning and hence better care for patients. However, formal communication and process structures are required to optimize the deployment, engagement, and professional development of clinical resources to better support chronic disease management and to avoid internal care fragmentation for more complex patients with clustered chronic conditions.

**Supplementary Information:**

The online version contains supplementary material available at 10.1186/s12875-023-02073-x.

## Introduction

Initially designed to address acute illness and injuries, the Canadian health care system needs to adapt to an aging population, in which the rising prevalence of chronic conditions demands a much greater focus on chronic disease management [1]. Two of the most common reasons for which older Canadians seek acute care are exacerbations of chronic conditions such as heart failure and chronic obstructive pulmonary disease, which are often compounded by dementia and frailty [2]. Initiatives to shift the Canadian system to a more proactive and preventative model of care have mostly focused on enhancing capacity in primary care [3, 4].

## Background

Over a decade ago, the Ministry of Health and Long-Term Care of Ontario, Canada’s most populous province, developed a policy framework based on Wagner’s Chronic Care [5] and Expanded Chronic Care Models [6] to encourage the redesign of health care practices to better support persons with chronic conditions [1]. The Ontario Chronic Disease Prevention and Management framework suggests that interprofessional and collaborative models of primary care can better support persons in managing their chronic conditions compared to traditional models, such as those based on purely Fee for Service physician remuneration [1]. Based on this framework, Ontario created primary care Family Health Teams to “enhance access to interprofessional, team-based care” and focus on the prevention and management of health and disease across the lifespan [3]. Funding for family health teams promoted the inclusion of multiple clinical professionals working to their full scope of practice, offered physicians flexible remuneration models, and accommodation for teams to shape clinic processes to meet the needs of their communities [7]. In addition, funding was provided for electronic medical records to improve care coordination and reporting of relevant quality and safety indicators [7].

The funding model and structure of Ontario family health teams aligns with several factors identified as necessary for successful chronic disease management in primary care, including care process reorganization to foster longitudinal and preventative care; engagement of interprofessional resources to increase the scope of practice and enhance patient-centered care planning; promotion of self-care through care coordination and education; implementation and use of electronic medical records; and development of frameworks and quality indicators to promote best practices [8]. Many family health teams have developed specific chronic disease clinics to facilitate better support and management of people with common chronic conditions [3]. Thus, the expectation from the Ministry was that family health teams would improve patient outcomes and patient experiences.

The effectiveness of Ontario family health teams has been mixed. An evaluation by the Conference Board of Canada reported that family health team patients experience similar quality of care and management of their chronic diseases as those served by other primary care clinic structures, yet patient satisfaction with care is high [3, 7]. A subsequent evaluation by the Institute for Clinical Evaluative Sciences suggests that while family health teams have fewer patients with high levels of comorbidity than those in traditional fee for service physician models, rates of emergency department utilization are often higher [9]. In addition, rates of hospitalization and rehospitalization at one year for those living with chronic conditions were lower than for community health centers (a salaried model of primary care) but higher than for fee-for-service models. Quality indicator ratings for diabetes care and cardiovascular prescribing were generally better in family health teams than in other care models.

Several barriers to developing effective family health teams have been identified in relation to interprofessional communication, collaboration, role clarity, and culture change [10–12]. A study of eight Ontario family health teams reported that their effectiveness could be enhanced through improved interprofessional collaboration between physicians and allied health clinicians, funding for competitive salaries, education about health professional roles and capabilities, and optimized clinical care processes [11]. Another review identified additional barriers to interprofessional primary care, including lack of role clarity and trust, hierarchical roles and relationships, inadequate communication tools, weak governance and leadership, financial disincentives related to remuneration, and lack of interprofessional education [13]. Failure to achieve effective interprofessional collaboration was identified as a key challenge to the effectiveness of family health teams [14].

One of the original Ontario family health teams developed several chronic disease management clinics, including a heart failure clinic, which was shown to improve optimal prescribing for patients with heart failure, increase referrals to cardiac rehabilitation, decrease symptom burden, and reduce hospitalizations by 68% over one year [15]. Successful heart failure management requires a high degree of interprofessional proficiency, which we hypothesized that this family health team had achieved [16]. Thus, our study objective was to understand how this family health team had approached the challenge of developing effective interprofessional chronic disease management clinics.

## Methods

### Design

An in-depth case study employing qualitative methods was conducted at a Southwest Ontario Family Health Team (henceforth referred to as the Family Health Team). The case study design was chosen for its ability to answer both “how” and “why” questions about the phenomenon when the context needs to be taken into consideration [17]. For this research project, the context of the Family Health Team was crucial in understanding and interpreting the collected data to understand how the team approached chronic disease management. Semi-structured individual interviews (see Supplementary File for Interview Guide) were conducted with health care professionals and other employees of the organization.

Ethics clearance for this case study was obtained through the *[Blinded for Review]* Office of Research Ethics (ORE#20774).

### Data Collection

Twenty-two individual semi-structured interviews were conducted in person in the clinic or by phone with various health care providers and other employees at the Family Health Team (Table 1). A letter of information was provided to staff at the family health team, and participants indicated if they would like to participate. The research team aimed to recruit a variety of clinical perspectives and roles and interviewed everyone who volunteered to participate. The interviews focused on the historical perspectives, development, and characteristics of the Family Health Team and its chronic disease management clinics. The interviewers aimed to explore the perspectives of the health care providers and support staff on the challenges and potential solutions for fostering interprofessional relationships and role clarity. Each interview was audio-recorded and transcribed verbatim and lasted between 30 and 60 minutes in length. The interviews were carried out by two research assistants (undergraduate and master’s-trained) and supervised by a co-investigator (PhD-trained - VB). The interviewers had no previous relationship with the interview participants. The senior author supports the family health team clinically and observed three interviews.

**Table 1:** Provides an overview of the interview participants, their role, and the length of time working in that specific position

Participant/Staff ID	Role	Time in Position
001	Nurse practitioner	5
002	Electronic medical record maintenance, quality assurance, quality improvement, data capturing/ standardization	Less than 1 year
003	Nurse Practitioner	10 months
004	Registered Nurse and lead nurse	13 years
005	Medical Doctor	10 years
006	Pharmacist	8 years
007	Registered Practical Nurse	14 years
008	Medical Doctor	On family health team since its inception
009	Registered Practical Nurse	7 years
010	Nurse Practitioner	Unknown
011	Medical Doctor	6.5 years
012	Nurse Practitioner	Unknown
013	Medical Doctor	Since its inception
014	Medical Doctor	5 years
015	Medical Doctor	1.5 years in memory clinic
016	Pharmacist	3 years
017	Medical Doctor- Cardiology	Unknown
018	Medical Doctor	Unknown
019	Former Nurse Practitioner	6 years
020	Medical Doctor	3.5 years
DH	Medical Doctor	Unknown
DF	Medical Doctor	Unknown
P	Pharmacist	6.5 years at family health team

### Data analysis

The transcribed data were analyzed inductively by three members of the research team (JE, LB, ZD^1^) in the software program NVivo 11. The researchers independently coded each of the 22 transcripts using initial line-by-line emergent coding, as described by Lofland et al. [18]. All transcripts from the interviews with employees and health care providers in the Family Health Team were included in the analysis and data saturation was obtained. The coded data were sorted into larger key themes through a process of focused coding based on consensus among a team of five researchers (JE, LB, ZD, VB, GAH) [18, 19]. The themes and their descriptions were discussed among the entire research team to agree upon an understanding of the overarching story emerging from the qualitative data [19]. A member check was conducted with the findings being shared with the clinical and administrative team of the Family Health Team.

## Results

Our analysis identified two main themes: [1] Interprofessional team building and [2] Inadvertent creation of silos.

### Theme 1. Interprofessional team building

We identified two subthemes within this theme: (a) Collegial learning; and (b) Informal and electronic communication.

#### Theme 1a. Collegial learning

A pre-existing culture of promoting leadership and teamwork led the Family Health Team to become one of the first family health teams in Ontario. Within this culture, healthcare providers stressed the importance of considering one another as colleagues and of sharing each other’s expertise as a vehicle towards the best possible care. One physician described the underlying rationale to create an environment in which physicians and allied health professionals would work with each other as teammates.

I think it was the idea of working with others to use their expertise in areas that they were better at. Some of the particular things we didn’t necessarily have the skill set for and some of the things we didn’t particularly want to do, and some of it quite frankly, was financially driven – (MD, 005)

The Family Health Team approached the development of interprofessional practice by explicitly asserting that all providers are equal partners collaborating to enhance patient care. Providers stressed how this dynamic fostered a more efficient distribution of work by encouraging nurses and other allied health workers to fully apply their scope of practice.

I think it’s a philosophical thing. When we first got the [nurse practitioners] and pharmacists here, I remember sitting with them and saying, ‘you can decide how you want to be seen here’ and ‘you can decide if you want to be seen as an employee or you could decide if you wanted to be seen as a colleague, and so you pick your path. If [...] you choose to be a colleague, then that will raise the bar for everybody’ – (MD, 008)

Intrinsic to this culture of fully deploying scopes of practice, new nursing, allied health, and physician staff were strongly encouraged to define their own roles within the Family Health Team and its clinics.

I think in large measure we were very much open to saying, well ‘tell us what you want to do’ ‘tell us how it would work’ and we weren’t very directive, and we were blessed by having really bright people at the beginning take that on. So we were very lucky to attract [nurse practitioners], pharmacist, dietician that were just on fire to develop these programs. – (MD, 008)

As a result, the broader education and health promotion needs of patients with chronic conditions were better met by the allied health professionals, providing other clinicians with greater capacity for timely assessment of more acutely ill patients. One nurse explained that this focus on management and education represented an important shift towards a more proactive approach to caring for people with complex conditions:

I think it’s a great thing and I have kind of seen the shift… from what I would call reactive to now proactive where we are assessing risk. We have an aging population, so obviously chronic diseases are going to be more prevalent [..], and so this affords us a chance to screen and weed out those patients that are at higher risk, and put measures in place - (RN, 004)

The flexibility inherent to this collegial culture imbued all providers with a sense of leadership and contributed to the development of chronic disease management clinics within the Family Health Team. These were developed one by one, starting with conditions for which management was amenable to relatively simpler care processes or algorithms, such as hypertension and diabetes. Beginning with such more ‘straightforward’ diseases allowed Family Health Team clinicians to focus on developing interprofessional relationships and defining clinic structures and roles.

Type 2 diabetics in particular had really piecemeal care depending on the individual physician [..]. A type 2 diabetic who could afford a nutritionist or afford a physiotherapist, got better care than somebody who was in the public system. So, we saw type 2 diabetes as something that we could easily do better than we were doing. And it was also the added benefit of nice markers that we could follow, you know, blood sugar, hemoglobin A1C, microalbumin. – (MD 008)

The commitment and efforts of allied health professionals to the success of the chronic disease management clinics not only led to better care for patients but also to the recognition by all clinicians of their value as agents of knowledge translation and dissemination. One physician explained how working alongside allied health professionals dedicated to managing a specific condition kept them informed and focused on current best practices:

I think it keeps us practicing better medicine when there are other people around taking an interest in the programs and the people that you work with. So when there is changes to chronic disease management, just simply by working in the same organization with the same people who are doing this disease management, helps me keep updated on what is happening in that field, better than if I sent them somewhere else. – (MD, 011)

As confidence with the interprofessional hypertension and diabetes clinics grew, the Family Health Team began to develop clinics for more complex conditions, including a pharmacist-managed anticoagulation clinic, a memory clinic, and a heart failure clinic. Each clinic was developed and operated under slightly different conditions and structures, primarily based on the nature of the disease and the clinician mix and interest.

#### Theme 1b. Informal and electronic communication

Upon its inception, the Family Health Team adopted a single electronic medical record to be shared by all members of the disciplinary team. This decision was initially controversial, as some allied professionals were accustomed to practicing with their own confidential chart for each patient. The adoption of a single chart aimed to enhance communication and care coordination:

The first meeting we had after approval of our team we made a decision… we are going to have [...] one patient, one chart. And the social workers found that quite alarming at first because to them, they had always the rights to the chart, there were private things in the social worker chart, and after all we wouldn’t want that shared amongst anybody. And we said no, we’re going to have one patient, one chart. Everybody has rights to it, and everybody shares in it. And so that was a bit of a paradigm shift for them, but I think it worked out in the course of the years... – (MD, 005)

Over time, providers agreed that having one chart per patient not only enhanced information sharing but also contributed to better patient care by allowing providers to gain a more holistic understanding of their patient. Moreover, the electronic medical record software in use at the Family Health Team also offers confidential and instant messaging to support communication, both synchronous and asynchronous, among individual clinicians.

So we do message each other as physicians and allied health as well as other team members all the time on our [electronic medical record]… I would say that’s one of our main methods of communication especially if we want something kind of recorded, even if it’s sometimes to say, ‘hey can I talk to you about this patient?’ - (MD, 011)

The physical layout of the Family Health Team both facilitated and hindered communication among clinicians. The Family Health Team itself consists of a large main site and a smaller satellite site elsewhere in the same municipality. While offered to all patients rostered with the family health team, all five chronic disease management clinics were located at the main site. Moreover, each site had separate servers hosting electronic medical records. Each server had a separate login process, and minimal patient information was shared beyond demographic information, thus creating communication and informational barriers among clinicians at different sites.

Within the main site, which hosted most of the allied health clinicians, most communication and information exchange was informal and ad hoc. Clinicians from the main site discussed how ‘water cooler’ conversations readily provided physicians, nurses, and other clinicians with opportunities to share updates on patient status, obtain clarifications on patient care, and request clinical advice. Considered a strength of the Family Health Team, clinicians acknowledged that such opportunities were not available to those at the satellite site:

The offices are very close to each other, everyone sees each other in the hallways, people are free to chat, say hello, or instant message or whatever, so the communication is easy. And I think that’s where the disadvantage for the [satellite] site is, they don’t have that same advantage that we do *– (MD, 005)*

Thus, while the implementation of a standard electronic medical record platform provided enhanced opportunities for formal information sharing, interprofessional communication remained primarily informal, putting the satellite site at a disadvantage both physically and electronically.

In summary, a culture of collegiality allowed physicians and allied health professionals to work towards their full scope of practice, setting conditions in place for the development of interprofessional chronic disease management clinics for increasingly complex conditions. A unique electronic medical record allowed for electronic information sharing, complemented by regular opportunities for informal communication. However, physical and electronic barriers impeded communication between clinicians from different sites of the Family Health Team. Structured and scheduled opportunities for interprofessional communication remained limited.

### Theme 2. Inadvertent creation of silos

The implementation of “in house” chronic disease management clinics within the Family Health Team was seen by some clinicians as allowing patients to receive detailed and specialized care for their complex conditions in a familiar setting while creating a more integrated and coordinated experience.

We saw it as a real advantage to have our patients see a team and have a multidisciplinary approach to their care. It was the first time, as family physicians, we had funding to be able to do that. Before that time, it was piecemeal, where you would have your patients see people in the community, in the hospital, you know... And this was really a gift to be able to centralize patient care in one location, under one roof. – (MD, 008)

In contrast, several other clinicians noted that the development of separate disease-specific chronic disease management clinics had led over time to the creation silos of care within the Family Health Team. A significant number of patients with multiple chronic conditions had been noted to be attending more than one clinic. For these patients, care coordination had actually become more complex and communication among individual clinics less effective, as one nurse explained:

“Certainly, the individual chronic disease programs there is a lot of communication within the members of the teams themselves, but we don’t always do such a wonderful job communicating it out to the rest of the [Family Health Team] team” - (RN, 004)

Such internal fragmentation was particularly problematic for the most responsible family physician. One physician explained feeling excluded and losing touch when patients were referred to these clinics:

So sometimes, I would see a patient months later and think, ‘oh I did that referral, what ended up happening’, and they were like ‘oh yeah I saw someone’, and I was like ‘oh you did’, and I wouldn’t have known. I would look back at copies of the test, but that was it - (MD, 011)

Suboptimal communication left some physicians concerned that the patient may not be receiving adequate care, particularly for other conditions falling beyond the scope of disease-specific chronic disease management clinics. One physician summarized the problem by explaining that providers in individual clinics were missing ‘the whole picture’.

…but I think the nice thing about family medicine is you get to see that patient as a whole, and sometimes in the chronic disease program we start just breaking it up into one thing, and all the rest of the patient’s health care kind of gets dropped, and the focus just ends up being on one little aspect of their medical care. (MD, 011)

Patients attending more than one clinic experienced redundant assessments. They were also required to book multiple appointments with different clinics, often on different days, which was seen as placing undue burden on them, and often also on their care partners:

Sometimes I find that my patients, especially seniors with multiple chronic diseases, then start kind of seeing this other doctor more than they see me, and sometimes it’s actually onerous on the patient to have to make these extra appointments. - (MD, 011)

Despite the creation of such internal silos, providers still considered that patients received better care through chronic disease management clinics, particularly for related comorbidities such as cardiovascular risk factors and conditions. This observation led to the idea of clustering chronic disease management clinics for related conditions to streamline care and reduce the burden on patients attending multiple clinics:

People that have hypertension have diabetes, and people that have hypertension and diabetes get heart failure, and people that get heart failure are the seniors in the population. [...] You know, the person is seen in the hypertension program, could they just go to one clinic and have all their needs met? Unfortunately, we built our chronic disease clinics as very separate entities and I think we did that, not necessarily purposefully, but to make it palatable at the time, and then we started to notice the same people are in all these clinics... – (NP003)

Thus, clinics focusing on common disease clusters could overcome the challenges from poor coordination and duplication of care stemming from the fragmented chronic disease management clinic structure while maintaining the benefits related to better patient management and education.

## Discussion

Family health teams were established in Ontario to improve chronic disease management and patient outcomes in primary care, but with minimal guidance on how to structure resources and support effective interprofessional collaboration and communication. Our case study of one of these family health team illustrates how emphasizing collegial, rather than hierarchical, relationships among all clinicians supported collaborative chronic disease management. By initially focusing on chronic disease management clinics for less complex conditions such as hypertension and diabetes, allied health clinicians were able to define and develop their roles and responsibilities in a family practice setting.

Within this family health team’s main site, staff proximity and electronic messaging facilitated informal communication and created shared learning opportunities within chronic disease management teams. However, these informal mechanisms were not always sufficient to support effective communication between individual clinics and between the main and satellite sites of the family health team, leading to the creation of care silos within the team itself. The introduction of clinics for more complex conditions further accentuated these unanticipated consequences, resulting in greater care fragmentation and undermining the intended benefits of the family health team.

There is increasing recognition that interprofessional collaboration is necessary to ensure optimal health outcomes for an ageing population of persons with increasingly complex health and social needs [20]. A survey of primary care practices in the province of Quebec, Canada, identified several factors associated with the provision of high-quality chronic disease care, including the presence of multiple health professions and a favourable interprofessional team climate [21]. Yet, the optimal approach to the provision of such care to older persons remains to be determined. A systematic review identified twenty-five different care models, over half of which were ineffective, and only five of which explicitly addressed interprofessional or multidisciplinary practice [22]. Our study findings show how emphasizing work-place collegiality allows professionals to define their clinical roles more clearly within a primary care practice, which is essential to ensure mutual accountability and provide quality care that meets patient needs [23, 24]. This approach is also consistent with recommendations from the Institute of Medicine report on the importance of integrating interprofessional learning with health service implementation [25]. This family health team explicitly embedded principles of collegiality and mutual accountability within its implementation of disease management clinics, likely contributing to better outcomes than observed in other Ontario family health teams [9, 11]. However, the question of which organizational structures are required for a primary care team to sustain effective interprofessional collaboration over time remains unanswered.

Our finding of internal care fragmentation within one family practice setting following the development of multiple clinics for complex chronic diseases is novel. In the aforementioned systematic review, most programs targeted a limited aspect of health promotion for patients with hypertension, diabetes, arthritis, pain and COPD, and few addressed the comprehensive care of patients with more complex conditions [22]. Primary care practices have finite resources with which they must support the needs of a wide spectrum of individuals from “cradle to death”. Internal care fragmentation results in the inefficient use of practice resources and thus not only affects patients with specific chronic diseases, but potentially all the other patients served by the practice. Our findings suggest that one solution to avoid this fragmentation is to integrate clinics for patients with conditions that cluster together, such as cardiovascular risk factors like hypertension and diabetes. Frailty, which stems from the accumulation of multiple physiologic deficits with age and increases vulnerability to stressors such as care fragmentation, is a health state amenable to chronic disease management principles [26, 27]. While no standard method to identify frailty exists, several brief case-finding approaches are easily implementable in a primary care setting [28, 29].

**Fig. 1:**
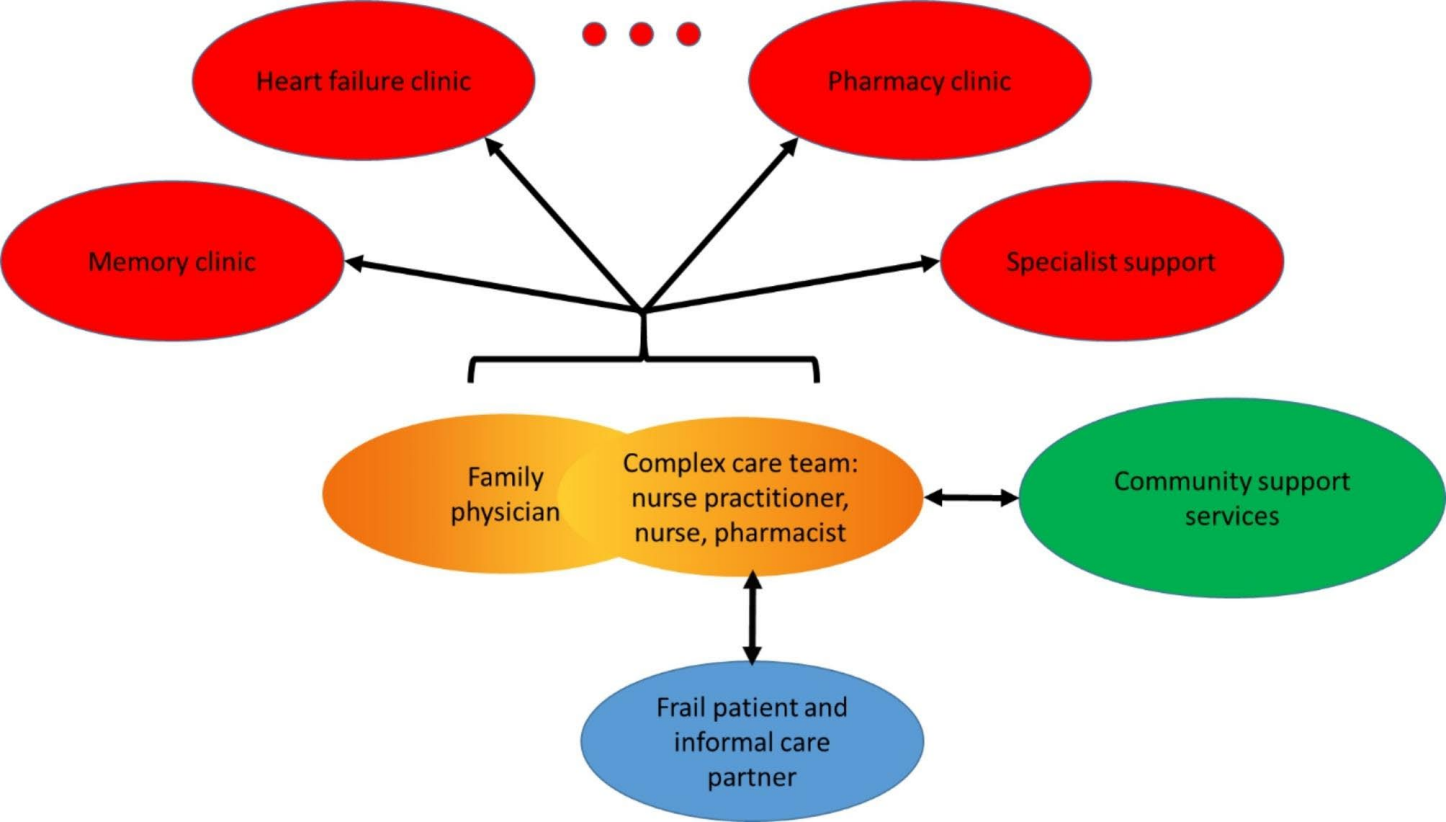
Proposed structure of a complex care program for frail persons

In response to our study’s findings, the Family Health Team developed a Complex Care Program for older adults with frailty and complex health problems, and that provides more formal communication between clinic team members and physicians [30]. This program uses a systematic case-finding approach, standardized assessment by an interprofessional team, and consultation with the primary care physician and a geriatrician. Additional condition-specific clinics may be involved (e.g., memory clinic), though case management remains coordinated within the Complex Care Program (Fig. 1). Preliminary data indicate that this program has led to greater involvement of allied health professionals with reduced overall health service utilization, self-management coaching for patients, improved prescribing and fewer falls, appropriate community support service referrals, and advance care planning discussions. Patient and provider satisfaction with the program is high. The program is currently being further evaluated through a grant from the Healthcare Excellence Canada Advancing Frailty Care in the Community initiative [31].

### Limitations

Several limitations to this study must be acknowledged. First, this study focused on the experience of one family health team with a specific set of clinical resources, and additional research is necessary to understand whether these findings reflect experiences in other primary care settings. However, the principles of interprofessional collaboration are transferrable to other settings and would in all likelihood support other Family Health Teams Moreover, the finding that formal communication processes are required to complement informal ones is likely also generalizable. Second, this study focused solely on the perspectives of clinicians in the family health team and not on patients or their caregivers. Ultimately, the success of interprofessional chronic disease care is best judged by recipients of care themselves. Third, while the outcomes of the heart failure clinic suggest that this family health team had achieved a high degree of competency in chronic disease management, these data are difficult to contextualize without comparable control groups. Nonetheless, the reported reduction in acute care utilization compares favourably to those demonstrated in the heart failure literature [32]. Fourth, while our approach to member checking was not directed at individual interviewees, presenting the results to the entire practice initiated a process leading to the development of an integrated clinical program for older persons with complex needs, an approach consistent with the principles of a learning health system [33]. Finally, our interviews did not explore issues related to the sustainability of optimal interprofessional practice, the conditions for which need ongoing support at the leadership and management level of an organization [34].

## Conclusion

Successful interprofessional chronic disease management clinics in primary care require a focus on collegial relationships, common workspaces, and both formal and informal communication processes. Integrated clinic settings based on structured case-finding and assessment of patients with related conditions, and integrated case-management supported by both formal and informal interprofessional communication, may provide more coordinated care to better serve vulnerable patients with complex needs. By optimizing the engagement, deployment, and professional development of clinical resources, we can support better patient outcomes.

## Electronic supplementary material

Below is the link to the electronic supplementary material.

Supplementary Material 1

## Data Availability

Despite being deidentified, given that the interviews were conducted at one of the original family health teams in Ontario, the transcripts could potentially reveal the identity of the persons interviewed. Thus, we will refrain from making these data available.

## Footnotes

1 Zachary DeVries was a co-op student who contributed to the analysis of the data.
