# Supplementary material for: Development, successes, and potential pitfalls of multidisciplinary chronic disease management clinics in a family health team: a qualitative study
Source: BMC Prim Care. 2023 Jun 20;24:126. doi: 10.1186/s12875-023-02073-x (PMC10280863; doi:10.1186/s12875-023-02073-x)
Supplement: Supplementary file 1 — Supplementary Material 1 [file 12875_2023_2073_MOESM1_ESM.pdf]

# Development, successes, and potential pitfalls of multidisciplinary chronic disease management clinics in a Family Health Team: A qualitative study.

## Supplemental file: Interview Guides

## APPENDIX G: RESEARCHER INTERVIEW GUIDE FOR ORIGINAL HCPs AT SOFHT

Participant ID: ___________________________ Data point: _____________________
Date: ___________________________ Recorder (RA):________________

Note:

The purpose of these individual, semi-structured interviews is to gather information on the historical perspectives of the SOFHT. These interviews are comprised of questions regarding care process challenges and solutions, the issue of establishing and fostering interprofessional relationships; the decision to move from a single co- morbidity, like hypertension and diabetes, to more chronic conditions, such as memory and heart failure. In addition, the interviews will invite participants' to describe their perspectives of past, current and anticipated sustainability concerns related to in-house expertise, training, funding, and acceptability among other practice physicians. Participants will be asked about health roles and role clarity of other health care professionals. The guide may require some modification based on data collected during preceding phases of the intervention.

**Introductions:**

Start with overview of study and interview objectives.
Explain consent and audio tape procedures.

Introduction**:** There are many concerns that need to be addressed in order to better manage and care for individuals who have chronic conditions. The SOFHT is a primary care provider who operates several Chronic Disease Prevention and Management (CDPM) clinics. The goal of the study is to conduct an in-depth case study of the CDPM clinics. We would like to hear from you about the historical perspectives of the SOFHT, taking into consideration the challenges and solutions that arose during the development of these clinics.

Start voice recorder (state date and interview number).

**Interview Questions:**

I would like to discuss your beliefs and perspectives to better understand your view on the SOFHT and CDPM clinics. I will ask you questions related to several topics to see how important these topics are to you.

Introduction Questions**:**

1. Can you describe SOFHT's approach to the CDPM clinics at the inception of the family health team (FHT)? How has this approach evolved over time? What events led to these changes?
   **Probes:** What worked? What failed?

2. Can you describe the interprofessional approach to care? How has the nature of interprofessional care and relationships evolved over time?
   **Probes:** Any ongoing concerns? Any factors for success?

Evidence:

3. Can you describe the quality of care that patients received prior to the changes made at the CDPM clinics?
   **Probes:** Did the quality of care change once these clinics were modified? If so, how?

4. Can you describe the quality of team work and communication before the changes were made at the CDPM clinics?
   a) Did team work and communication change once the modifications had been made?
      **Probes:** In what ways did it improve? In what ways did it worsen?

5. Of all the changes made to the SOFHT (e.g., shifting focus from single co-morbidities to more complex conditions, establishing interprofessional relationships, etc.), what change has been the most valuable to the team and patients?

6. Do you feel that team members/clinicians accepted the CDPM clinics?
   **Probes**: Did they buy into it? Did they seem reluctant to the clinics?

7. How do you feel managers' and administrators' practices have influenced the changes made to the SOFHT and CDPM clinics?
   **Probes:** What practices do you think have been most beneficial in influencing the CDPM clinics? The most detrimental?

8. Can you talk a bit about the role you had in identifying, developing, and implementing changes to the SOFHT and the CDPM clinics? Are you happy with the role you've had in this process and with the final changes that were made?

9. How have the changes to the CDPM clinics impacted patients' primary care?**Probes**: Beneficial impacts? Detrimental impacts?

10. What were some of the challenges associated with introducing the clinics to help manage chronic conditions?
    a) What impact did these clinics have on information collection? On overloads? On streamlining?
    b) How did the clinics affect the care provided to the patients?
    c) How did the clinics affect team work and communication?
       **Probes:** Communication between staff? Between staff and patients? Between patients and their families?

<u>Context:</u>

11. What factors within your clinical environment assisted with making the needed changes to the CDPM clinics?

a) How did these factors assist you with making needed changes to the CDPM clinics?
b) Are you able to do more/ better with what you have, now that the clinics are in place?
c) What kinds of efficiencies could be achieved by redesigning the care processes?
**Probes**: Time? Resources?

12. In regards to being a part of an interprofessional team:
    a) Are you able to do more/better?
    **Probes:** How has it impacted the care of patients in the CDPM clinics? How has it impacted communication and team work?
    b) What are some challenges?
    **Probes:** How has it impacted the care of patients in the CDPM clinics? How has it impacted communication and team work?

Facilitation:

13. In regards to other health care professionals:
    a) Describe your past, current and anticipated concerns of other health professionals' in-house expertise, training, funding, and acceptability.
    b) Have these concerns changed over time?
    c) Have the modifications to the CDPM clinics improved or worsened your concerns?

14. What else can be changed in regards to the SOFHT or CDPM clinics?
    a) What do you think will improve primary care of patients with chronic diseases?
    b) How can this be implemented within these clinics?

**Closing notes**

I would like to thank you for your input and perspective. Are there any other comments that you would like to make or relevant stories that you would like to share?

Thank you.

**APPENDIX H: RESEARCHER INTERVIEW GUIDE FOR CURRENT HCPs AT SOFHT**

Participant ID: ___________________________ Data point:_ ___________________
Date: ____________________________ Recorder (RA):________________

Note:

The goal of these individual, semi-structured interviews is to gather information on the physicians' perspectives about access to Chronic Disease Prevention and Management (CDPM) clinics, the impact that these clinics have on patients' health, resources, and achieving balance, in terms of the distribution of internal resources, communication with clinic staff, and potential conflicts or disagreements with non- lead/specialty clinic physician(s). Their perspectives on other health care professionals' roles and role clarity, learning, support needs, and integration with external specialists or other programs will also be discussed. Lastly, their comfort level with managing chronic conditions, and patients with chronic conditions in an interprofessional, primary care setting will be discussed. The guide may require some modification based on data collected during preceding phases of the intervention.

**Instructions:**
Start with overview of study and objectives of the interviews.
Explain consent and audio tape procedures.

Introduction: There are many concerns that need to be addressed in order to better manage and care for individuals with chronic conditions. The SOFHT is a primary care provider who has several CDPM clinics. The goal of the study is to conduct an in-depth case study of the CDPM clinics. We would like to hear from you about your perspective of these clinics.

Start voice recorder (state date and interview number).

**Interview Questions**
I would like to discuss some of your beliefs and perspectives to better understand your view on the SOFHT and CDPM clinics. I will ask questions related to several topics to see how important they are to you.

Introduction Questions:

1. Can you tell me what you think about the SOFHT and CDPM clinics?

2. Overall, what has your experience been like while working at the SOFHT?

Evidence:

3. Can you describe the quality of care that patients receive from the CDPM clinics? Has this changed since you started your position at the SOFHT? If so, how?
   **Probes:** What are some positives about the quality of care that they receive? Negatives?

4. Can you describe the quality of team work and communication? Has this changed since you started your position at the SOFHT? If so, how?
   **Probes:** What are some positives about the team work and communication? Negatives?

5. How confident are you in the clinics and the role that they have?
   **Probes**: How do they affect patient care? How do they affect team work and communication?

6. How would you describe patients' level of access to the CDPM clinics?
   **Probes**: In what ways is the access easy? In what ways is it challenging?

7. How do you feel the managers' and administrators' practices within the SOFHT have influenced the CPDM clinics?
   **Probes:** What practices do you think have been most beneficial in influencing the CDPM clinics? Most detrimental?

8. Have the CDPM clinics affected your workload? If so, how?
   **Probes:** Are you faced with too little or too much work now?

9. How comfortable do you feel managing patients with chronic conditions? How comfortable are you with managing these individuals within an interprofessional, primary care setting?
   a) Do the CDPM clinics affect your comfort level with managing these patients? How?
      **Probes:** Do the CDPM clinics aid? Facilitate? Interfere?
   b) What things could help improve your comfort level with managing these patients?

<u>Context:</u>

10. In regards to the roles that other health care professionals have within the CDPM clinics:
    a) Which of these roles make your job harder to carry out?
    b) Which of these roles make your job easier to carry out?
    **Probes:** Why?

11. What factors within your clinical environment assist with the implementation of the CDPM clinics?
    **Probes:** How do these factors contribute?

12. Think about the way that the SOFHT communicates with other health care professionals, external specialists, or other programs/clinics.
    a) Do you think that the communication between your team and other teams/ programs is successful?
    b) What could improve the communication?
    c) Have there been any conflicts in the past? Are there any conflicts now?

<u>Facilitation</u>

13. What else can be changed in regards to the SOFHT or the CDPM clinics?
    a) What do you think will improve primary care of patients with chronic conditions?
    b) How can this be implemented within these clinics?

**Closing notes**
I would like to thank you for your input and perspective. Are there any other comments you would like to make or relevant stories you would like to share?

Thank you.

**APPENDIX I: RESEARCHER INTERVIEW GUIDE FOR CURRENT PHARMACISTS, DIETICIANS AND NPs**

Participant ID: ___________________________ Data point: _____________________
Date: ____________________________ Recorder (RA):_________________

Note:

The goal of these individual, semi-structured interviews is to gather information on these individuals' perspectives about their clinical role and enactment, as well as how their role is perceived by other team members. Participants will be asked about their potential to expand their role and the barriers that they may face when doing so. Lastly, participants will be asked how comfortable they are with managing chronic conditions. The guide may require some modification based on data collected during preceding phases of the intervention.

**Instructions:**

Start with overview of study and objectives of interviews.
Explain consent and audio tape procedures.

Introduction: There are many concerns that need to be addressed in order to better manage and care for individuals with chronic conditions. The SOFHT is a primary care provider who has several Chronic Disease Prevention and Management (CDPM) clinics. The goal of the study is to conduct an in-depth case study of the CDPM clinics. We would like to hear from you about your perspectives in regards to these clinics.

Start voice recorder (state date and interview number).

**Interview Questions**

I would like to discuss some of your beliefs and perspectives to better understand your view on the SOFHT and CDPM clinics. I will ask questions related to several topics to see how important they are to you.

Introduction Questions:

1. Can you tell me what you think about the SOFHT and CDPM clinics?

2. Overall, what has your experience been like while working at the SOFHT?

Evidence:

3. Can you describe the quality of care that the patients receive? Has this changed since you started your position at the SOFHT? How?
   **Probes:** What are some positives about the care received? Negatives?

4. Can you describe the quality of team work and communication? Has this changed since you started your position at the SOFHT? How?
   **Probes:** What are some of the positives about the team work and communication? Negatives?

5. Can you describe your experiences with the patients at the MAIN site? Can you describe your experiences with the patients at the SATELLITE site?
   **Probes:** How are your experiences the same at each site? How are they different?

6. What are the current care processes, in terms of patient volume, program organization, staffing, requirements, resource allocations, and patient load at the SOFHT?
   **Probes:** How are these beneficial? How could they be improved?

7. What is your clinical role within the SOFHT?
   a) How does this role benefit your team and the CDPM clinics?
   b) How do you think you could improve your current role?
      **Probes:** What would help you improve your role?
   c) How would others within the SOFHT describe the role that you carry out?**Probes:** What benefits do you think they see your role as having? Detriments?

8. How do you feel team members' practices at the facility influence the CDPM clinics?
   **Probes:** What practices do you think have been most beneficial in influencing the CDPM clinics? Most detrimental?

9. How do you feel the managers' and administrators' practices influence the CDPM clinics?
   **Probes:** What practices do you think have been most beneficial in influencing the CDPM clinics? Most detrimental?

10. How comfortable do you feel with managing patients with chronic conditions?
    a) Do the CDPM clinics affect your comfort level with managing these patients? How?
       **Probes:** Do the CDPM clinics aid? Facilitate? Interfere?
    b) What things could help improve your comfort level with managing these patients?

11. Can you talk a little about your role within the CDPM clinics? Are you happy with the role that you have?
    a) Does this role help contribute to the effectiveness of the SOFHT? Of the CDPM clinics? Of the care of the patients? If so, how?
    b) How do you think you could improve your role to help with the effectiveness of the CDPM clinics?

<u>Context:</u>

12. What factors within your clinical environment assist with the implementation of the CDPM clinics?
    **Probes:** How do these factors contribute?

13. What support needs are included at the SOFHT to help you carry out your role?
    **Probes:** Which employees provide these supports? How do these supports help? Are there any other supports that you would like to have?

Facilitation:

14. In regards to your role within the SOFHT:
    a) Do you feel that you are capable of expanding your role?
    b) Would you say that one of your goals is to expand your role?
    c) What factors do you think are able to help you expand your role; what factors may prove to be barriers when trying to expand your role?

15. What else can be changed in regards to the SOFHT and CDPM clinics?
    a) What do you think will improve primary care of patients with chronic conditions?
    b) How can that be implemented within the SOFHT?

**Closing notes**

I would like to thank you for your input and perspective. Are there any other comments you would like to make or relevant stories you would like to share?

Thank you.

## APPENDIX J: RESEARCHER INTERVIEW GUIDE FOR HEALTH OUTCOMES EVALUATORS

Participant ID: ___________________________ Data point: ____________________
Date: ____________________________ Recorder (RA):________________

Note:

The goal of these individual, semi- structured interviews is to gather information on the suitability and shortcomings for current electronic medical record platforms, regarding data collection and quality assurance. This will include either embedding quality indicators for various programs or an interRAI- based solution that, with programming, could facilitate more integrated information capture and produce derivative outputs such as risk scores, outcome scales, other quality indicators and case- mix data. The guide may require some modification based on data collected during preceding phases of the intervention.

**Instructions:**
Start with overview of study and objectives of interviews.
Explain consent and audio tape procedures.

Introduction: There are many concerns that need to be addressed in order to better manage and care for individuals who have complex, chronic conditions. The SOFHT is a primary care provider who has several Chronic Disease Prevention and Management (CDPM) clinics. The goal of the study is to conduct an in-depth case study of the CDPM clinics. We would like to hear from you about the information technology issues that exist at the SOFHT.

Start voice recorder (state date and interview number).

**Interview Questions**
I would like to discuss some of your beliefs and perspectives to better understand your view on the SOFHT and CDPM clinics. I will ask questions related to several topics to see how important they are to you.

Introduction Questions:

1. Can you tell me what you think about the SOFHT and CDPM clinics?

2. Overall, what has your experience been like while working at the S0FHT?

Evidence:

3. How were the previous electronic medical record platforms?
    a) When changes were made, how did this affect data collection and quality assurance?**Probes:** In what ways were these changes beneficial? Detrimental?

4. How have the electronic medical record platforms at the SOFHT helped with the care of patients within the CDPM clinics?
   **Probes:** Has the care that they received been improved? Worsened?

5. Do you notice any major changes within the SOFHT, CDPM clinics, and/ or with the care of patients when the electronic medical record platforms change?

6. How are the current electronic medical record platforms suitable in regards to data collection and quality assurance?

7. Can you talk a bit about your role on the SOFHT?
   a) Are you happy with the role that you have?
   b) How does this role help contribute to the effectiveness of the: SOFHT, CDPM clinics and care of the patients?

Context:

8. What factors within your clinical environment assist with the implementation of the CDPM clinics?
   **Probes:** How do these factors contribute?

9. What physical environment changes have been implemented within the CDPM clinics since you began your position at the SOFHT?
   a) How have you approached these changes?
   b) In what ways have these changes been beneficial? Detrimental?
   c) Are there any other changes that you think need to be made? If so, why?

10. Are there any factors that make it difficult for you to carry out your job? Explain. **Probes:** People? Communication? Environment?

Facilitation:

11. What are some shortcomings within the current electronic medical record platforms, in terms of data collection and quality assurance?
    a) How could these be changed in order to overcome them?
    b) Out of all the shortcomings that currently exist in regards to the electronic medical records platforms, what do you think needs attention first?
       **Probes:** Why? How would you make these changes?

12. Think about the current electronic medical record platforms.
    a) Which quality indicators do you think would be the most important to focus on?
       **Probes:** Why?
    b) How would you embed quality indicators into the platforms for the various CDPM clinics?
    c) How would you tackle the differences in quality indicators needed for the varying CDPM clinics?

13. Think about the electronic medical records platforms.
    a) How would you implement interRAI instruments?
    b) What could be potential benefits to this? What could be potential risks?

14. What else do you think can be changed in regards to the SOFHT and/or CDPM clinics?
    a) What do you think will lead to better primary care of patients with chronic diseases?
    b) How can this be implemented within the SOFHT?

**Closing notes**

I would like to thank you for your input and perspective. Are there any other comments you would like to make or relevant stories you would like to share?

Thank you.

## APPENDIX K: RESEARCHER INTERVIEW GUIDE FOR SATELLITE HCPs

Participant ID: ____________________________ Data point: _____________________
Date: _____________________________ Recorder (RA):________________

Note:

The goal of these individual, semi-structured interviews is to gather information on these physicians' perspectives in terms of access and effectiveness of the clinics that are given off- site. The potential downfalls and problems related to communication and shared care will be discussed as well as the potential benefits compared to the usual approaches. They will also discuss perspectives on other health care professionals' roles and role clarity, improvements, and comfort level with managing complex conditions. The guide may require some modification based on data collected during preceding phases of the intervention.

**Instructions:**
Start with overview of the study and objectives of the interviews.
Explain consent and audio tape procedures.

Introduction: There are many concerns that need to be addressed in order to better manage and care for individuals who have complex, chronic conditions. The SOFHT is a primary care provider who has several Chronic Disease Prevention and Management (CDPM) clinics. The goal of the study is to conduct an in-depth case study of the CDPM clinics. We would like to hear from you about your personal perspectives regarding the SOFHT and CDPM clinics.

Start voice recorder (state date and interview number).

**Interview Questions**
I would like to discuss some of your beliefs and perspectives to better understand your view on the SOFHT and CDPM clinics. I will ask questions related to several topics to see how important they are to you.

Introduction Questions:

1. Can you tell me about what you think about the SOFHT and CDPM clinics?

2. Overall, what has your experience been like while working at the SOFHT?

Evidence:

3. Can you describe the quality of care that the patients receive?
    a) Has this changed since you started your position at the SOFHT? If so, how?
    **Probes:** Have the changes proven to be beneficial? Harmful? How?

4. Do you think the quality of care that the patients receive within the CDPM clinics, at the SATELLITE site; is the same as the care received at the MAIN site?

**Probes:** How is it the same? How is it different?

5. How well do you feel that you, and your team, are integrated with the SOFHT at the MAIN site?
    a) What are some benefits about the integration between the two different sites? Cons?
    b) Are there any success stories you can share about the integration between the two sites?
    c) Are there any problems?
    **Probes:** Are these problems short-term? Long-term? What do you think could help solve the problems?

6. Think about the effects that unconnected electronic medical records have, between the two sites.
    a) How can this be beneficial?
    b) How can this be problematic?
    c) How can the challenges be improved?

7. What is your clinical role within the SOFHT?
    a) How does this role benefit your team and the CDPM clinics?

8. How comfortable do you feel with managing patients with complex, chronic conditions?
    a) Do the CDPM clinics affect your comfort level with managing these patients? How?
    **Probes:** Do the CDPM clinics aid? Facilitate? Interfere?
    b) What things could help improve your comfort level with managing these patients?

Context:

9. What factors within your environment at the SOFHT assist in your role?
    a) How do these factors affect the role that you carry out?
    **Probes:** How does the environment positively affect your role? Negatively?

10. Are there any factors that make it difficult for you to carry out your job?
    **Probes:** Employees? Managers/bosses? Environment? Communication?

11. Are there any barriers to the access and effectiveness of the CDPM clinics at the SATELLITE site?
    **Probes:** Are you able to access every clinic for all of the patients? Do you rely on specialists/ external individuals in order to access the clinics for all patients? Is the access that you have at the SATELLITE site, different than the MAIN site?

12. What are some of the potential downfalls and problems that you see, as a physician at the SATELLITE site, in terms of communication and shared care?
    a) What changes do you think should be made in order to overcome the downfalls and problems? How would you implement the needed changes?

Facilitation:

13. What do other health care professionals do to help facilitate the changes in the SOFHT and CDPM clinics?
    **Probes:** In what way do they make a difference?

14. What can be changed in regards to the SOFHT and CDPM clinics?
    a) What do you think will improve primary care of patients with chronic diseases?
    b) How can that be implemented within the SOFHT?

**Closing notes**

I would like to thank you for your input and perspective. Are there any other comments you would like to make or relevant stories you would like to share?

Thank you.

**APPENDIX L: RESEARCHER INTERVIEW GUIDE FOR HEALTH CARE PARTNER ORGANIZATIONS**

Participant ID: ___________________________ Data point: ____________________

Date: ___________________________ Recorder (RA):________________

Note:

The goal of these individual, semi-structured interviews is to gather information on the benefits and concerns regarding overall care as well as system benefits and ongoing communication challenges. Lastly, participants will be asked to reflect on primary care provided to patients in other settings. The guide may require some modification based on data collected during preceding phases of the intervention.

**Instructions:**

Start with overview of study and objectives of interviews.
Explain consent and audio tape procedures.

Introduction: There are many concerns that need to be addressed in order to better manage and care for individuals who have complex, chronic conditions. The SOFHT is a primary care provider who has several Chronic Disease Prevention and Management (CDPM) clinics. The goal of the study is to conduct an in-depth case study of the CDPM clinics. We would like to hear from you about your personal perspectives regarding overall care, primary care in varying settings, and communication challenges.

Start voice recorder (state date and interview number).

**Interview Questions**

I would like to discuss some of your beliefs and perspectives to better understand your view on the SOFHT and CDPM clinics. I will ask questions related to several topics to see how important they are to you.

Introduction Questions:

1. Can you tell me what you know about the SOFHT and CDPM clinics?

2. Can you tell me what you think about the LOCAL General Hospital (LGH) heart failure (HF) clinic?

Evidence:

3. What is your role at LGH HF clinic?
   a) Are you happy with the role that you carry out?
   b) How does your role contribute to the care of patients?
      **Probes:** How does it improve the care of patients? How could you improve your role to better care for the patients?

4. How familiar are you with the SOFHT in general?
   a) How familiar are you with the SOFHT CDPM clinics, specifically the HF clinic?
   b) To your understanding, how and why did the CDPM HF clinic develop and evolve?

5. How do you perceive the quality of care of the patients with HF in primary care in general?
   a) What are some of the positives in regards to the quality of care received?
   b) What are some of the issues in regards to the quality of care received?
   c) How does the quality of care that is received affect the patients?
   d) How does the quality of care that the patients receive affect the operation of LGH HF clinic, specifically?
      **Probes:** How does it affect: patient load, ability to discharge, communication?

6. How would you compare the quality of care of HF patients at the SOFHT to other primary care settings?
   **Probes:** How would you compare patient outcomes? Care processes? Communication?Ongoing challenges?
   a) How is the quality of care different? How is it the same?
   b) Are there any benefits of one over the other? If so, what?

<u>Context:</u>

7. What are some of the potential downfalls and problems that you see in terms of communication and shared care?
   a) How do you think these can be improved?

<u>Facilitation:</u>

8. How should a specialized clinic, like the LGH HF clinic, support primary care?

9. What primary care supports are required to work more effectively with the HF clinic to improve patient care?

**<u>Closing notes</u>**

I would like to thank you for your input and perspective. Are there any other comments you would like to make or relevant stories you would like to share?

Thank you.
